# Ingesting a small amount of beer reduces arterial stiffness in healthy humans

**DOI:** 10.14814/phy2.13381

**Published:** 2017-08-07

**Authors:** Masato Nishiwaki, Naoki Kora, Naoyuki Matsumoto

**Affiliations:** ^1^ Faculty of Engineering Osaka Institute of Technology Osaka Japan; ^2^ Faculty of Environmental Symbiotic Sciences Prefectural University of Kumamoto Kumamoto Japan

**Keywords:** Alcohol, arterial stiffening, arteriosclerosis, cardio‐ankle vascular index, pulse wave velocity

## Abstract

Epidemiological studies reveal a J‐shaped association between alcohol consumption and arterial stiffness, with arterial stiffening lower among mild‐to‐moderate drinkers than heavy drinkers or nondrinkers. This study aimed to examine the effects of ingesting a small amount of beer, corresponding to the amount consumed per day by a mild drinker, on arterial stiffness. Eleven men (20–22 years) participated, in random order and on different days, in four separate trials. The participants each drank 200 or 350 mL of alcohol‐free beer (AFB200 and AFB350) or beer (B200 and B350), and were monitored for 90 min postingestion. There were no significant changes in arterial stiffness among trials that ingested AF200 or AF350. However, among trials ingesting B200 and B350, breath alcohol concentrations increased significantly, while indexes of arterial stiffness decreased significantly for approximately 60 min: carotid‐femoral pulse wave velocity (B200: −0.6 ± 0.2 m/sec; B350: −0.6 ± 0.2 m/sec); brachial‐ankle pulse wave velocity (B200: −53 ± 18 cm/sec; B350: −57 ± 19 cm/sec); and cardio‐ankle vascular index (B200: −0.4 ± 0.1 unit; B350: −0.3 ± 0.1 unit). Furthermore, AFB showed no effect on arterial stiffness, regardless of whether or not it contained sugar, and no significant difference in antioxidant capacity was found between AFB and B. This is the first study to demonstrate that acute ingestion of relatively small amounts of beer reduces arterial stiffness (for approximately 60 min). Our data also suggest that the reduction in arterial stiffness induced by ingestion of beer is largely attributable to the effects of alcohol.

## Introduction

Increased arterial stiffness has been identified as an independent risk factor for future cardiovascular disease (Laurent and Boutouyrie 2007). Pulse wave velocity (PWV) is generally used as an index of arterial stiffness. Large elastic artery stiffness is progressively greater with advancing age, even in healthy people (Avolio et al. 1985), but physical activity and dietary habits can alter the severity of the age‐related arterial stiffening (Tanaka and Safar 2005). Interestingly, epidemiological studies have demonstrated a J‐shaped association between alcohol intake and arterial stiffness (Sierksma et al. 2004; Hougaku et al. 2005; Mattace‐Raso et al. 2005): arterial stiffness is significantly lower in mild‐to‐moderate drinkers than in both nondrinkers and heavy drinkers (Sierksma et al. 2004; Hougaku et al. 2005; Mattace‐Raso et al. 2005). These epidemiological studies suggest the possibility that habitual mild‐to‐moderate alcohol consumption might reduce or prevent arterial stiffening.

Beer is the most widely consumed alcoholic beverage in the world, and contains not only alcohol but also, like red wine, polyphenols (Arranz et al. 2012; de Gaetano et al. 2016). A number of studies have noted the vascular protective effects of beer (Toda and Ayajiki 2010; Arranz et al. 2012; de Gaetano et al. 2016; Zhou et al. 2016), but few of them addressed the question of whether beer ingestion reduces arterial stiffness, so this issue remains a matter of debate (Krnic et al. 2011; Karatzi et al. 2013). Epidemiological studies have classified mild‐to‐moderate beer consumption as approximately 350–3500 mL per week (Sierksma et al. 2004; Hougaku et al. 2005; Mattace‐Raso et al. 2005), but in previous experimental studies, subjects have consumed 400–500 mL per day, which approaches the upper limit of the daily limit for the mild‐to‐moderate drinker classification (Krnic et al. 2011; Karatzi et al. 2013). If regular beer ingestion at mild‐to‐moderate levels actually reduces or prevents arterial stiffening, ingesting even a small amount of beer (350 mL or less per day) should measurably reduce arterial stiffness. However, as far as we can ascertain, no study has attempted to investigate the acute effects of ingestion of 350 mL or less of beer on arterial stiffness.

In addition to the quantity of beer ingested, three other major issues must be addressed. First, in prior experimental studies, subjects were usually given food (e.g., sandwiches) along with the test beverage (Karatzi et al. 2013; Fantin et al. 2016); but acute food intake exerts strong effects on PWV reduction (Ahuja et al. 2009; Taylor et al. 2014), so it is unclear whether previous findings were actually related to beer per se, or rather, to food or to the combination of food and test drinks (Karatzi et al. 2013). Second, beer contains antioxidant substances (i.e., polyphenols) (Arranz et al. 2012; de Gaetano et al. 2016); thus the control drink should not be water, but rather, dealcoholized‐beer that contains the same antioxidant substances as beer. Moreover, because there are the two types of dealcoholized‐beers with or without sugar, it is important to examine the effects of ingesting sugars in dealcoholized‐beers on arterial stiffness. Third, subjects in the previous studies typically consumed 400–500 mL of water along with the 400–500 mL of beer (Krnic et al. 2011; Karatzi et al. 2013), but such protocols can introduce unintended effects: ingesting a lot of fluid, such as a single liter, can cause changes in arterial stiffness and should be avoided. To our knowledge, no data are available concerning whether acute beer ingestion independently reduces arterial stiffness after considering the influences.

We hypothesized that ingesting only a small amount of beer (corresponding to the lower daily limit of a mild‐to‐moderate drinker) would reduce arterial stiffness. The primary purpose of this study, therefore, was to elucidate the acute effects of ingestion of a small amount of beer on arterial stiffness.

## Materials and Methods

### Participants

Eleven healthy young males participated in this study. The mean age, height, body mass, body mass index (BMI), and body fat of all participants were 21.1 ± 0.2 years, 170.3 ± 1.5 cm, 62.6 ± 2.6 kg, 21.5 ± 0.7 kg/m^2^, and 18.9 ± 1.6%, respectively. No participants had chronic diseases that could affect cardiovascular health, metabolism, or daily physical activity; none had history of smoking; and none were taking any medications. They all habitually consumed alcohol‐containing beverages (4.2 ± 1.5 g alcohol/day on 1.5 ± 0.7 days/week), but none exceeded the recommended amount of alcohol (40 g/day) beyond which there is increased risk of developing lifestyle‐related diseases in Japan. The purpose, procedures, and risks of the study were explained to each participant. All participants provided written informed consent before participating in the study, which was reviewed and approved by the Human Ethics Committee at the Osaka Institute of Technology (approval number: 2016‐5) and developed in accordance with the guidelines of the Declaration of Helsinki.

### Experimental procedures

All experiments were conducted in a quiet air‐conditioned room (22–24°C). To avoid potential diurnal variations, for each participant, all experimental sessions were conducted at the same time of day and the same number of hours after a light meal (at least 4 h). All participants were asked to abstain from alcohol‐ and caffeine‐containing beverages and to avoid strenuous physical activity for 12 h prior to an experimental session. In addition, participants were advised to eat the same meals (breakfast, lunch, and dinner) on the day before each experimental session.

**Figure 1:**
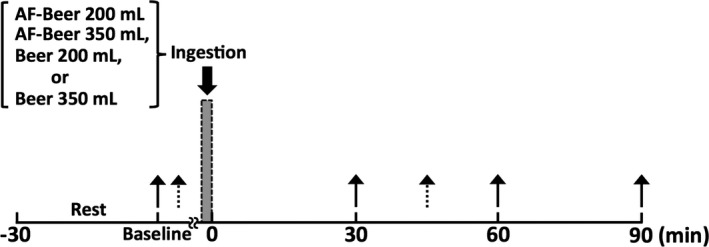
Time course of the experiment. Solid arrows, time points of measurements of breath alcohol level, brachial‐ankle pulse wave velocity, cardio‐ankle vascular index, and hemodynamics; broken arrows, time points of measurements of carotid‐femoral pulse wave velocity; AF, alcohol‐free.

The time course of the experiment is presented in Figure 1. All volunteers participated in four trials assigned in random sequence to four separate days: (1) 200 mL of alcohol‐free beer (AF200); (2) 350 mL of alcohol‐free beer (AF350); (3) 200 mL of beer (B200); and (4) 350 mL of beer (B350). This meant that each participant consumed 3.3 ± 0.1 mL of beer/kg body mass in B200 and 5.7 ± 0.2 mL of beer/kg body mass in B350.

On days 1 through 4, participants arrived at the laboratory and rested for at least 30 min, after which breath alcohol concentration (BAC), heart rate (HR), blood pressure (BP), and PWV were assessed to establish a preingestion baseline. Participants then consumed test drinks within 5 min, without any food. We used commercially available beer (B; The Premium Malt's, Suntory Holdings, Osaka, Japan) and alcohol‐free beer (AF; All‐Free, Suntory Holdings, Osaka, Japan) as test and control drinks, respectively. The beer was 5.5% alcohol by volume and the alcohol‐free beer contained no alcohol. All of the test drinks were poured into paper cups to maintain participant blinding (i.e., single‐blind study). After consuming a test drink, each participant rested on a comfortable chair for 90 min while the biometric measurements were repeated at 30, 60, and 90 min postingestion.

### Assessment of each parameter

The same investigators performed all measurements. Assessments of BAC were made in triplicate using a breath alcohol detector (AL‐1; Daiji Industry, Osaka, Japan), and the mean value was used for analysis. According to a previous study (Hirayanagi et al. 2006), circulating alcohol levels were also estimated using the following formula; circulating alcohol levels (%) = BAC (mg/L)/5. HR, BP, and PWVs were measured using a semi‐automated device (VS‐1500AE/AN; Fukuda Denshi, Tokyo, Japan) with participants in supine position, as described in our previous studies (Nishiwaki et al. 2014, 2015, 2017). Cuffs to measure BP and PWV were wrapped around both brachial upper arms and ankles, and then brachial‐ankle PWV (baPWV) and cardio‐ankle vascular index (CAVI) were obtained as indexes of arterial stiffness. In seven of the participants, carotid‐femoral PWV (cfPWV), which is an index of central arterial stiffness, was also measured with the same device, both at baseline and 45 min postingestion. Carotid and femoral arterial pressure waveforms were recorded by amorphous pulse wave sensors (TY‐501A; Fukuda Denshi) attached to the left common carotid and left common femoral arteries; values were automatically calculated as the distance between the carotid and femoral artery sites divided by the transit time. In our laboratory, coefficients of variation across observers' measurements, over ten subjects on two separate days (for reproducibility), were 7.5 ± 1.2%, 2.7 ± 0.3%, and 3.6 ± 0.6% for cfPWV, baPWV, and CAVI, respectively (Nishiwaki et al. 2014, 2015, 2017).

### Supplementary experiments

Beer generally contains sugar, but the alcohol‐free beer used as the control drink in our study was also free of sugars. Therefore, in Supplementary Experiment 1, we investigated the effects of ingesting sugars in beer on arterial stiffness by giving participants either alcohol‐free beer with sugars (experimental group) or without (control group). Five participants consumed 350 mL each (in separate experimental sessions) of alcohol‐free, sugar‐containing beer (AFS; Kirin Free, Kirin Brewery, Tokyo, Japan) and alcohol‐free, sugar‐free beer (AF). The experimental procedures were the same as for the main study, and BAC, baPWV, and CAVI were assessed at baseline and at 30, 60, and 90 min postingestion.

In Supplementary Experiment 2, to determine antioxidant capacity in the each test drink, 1,1‐Diphenyl‐2‐picrylhdrazyl (DPPH) free radical scavenging activity was assessed using a spectrophotometric analyzer (DU 800, Beckman Coulter, Tokyo, Japan) in a dark room (Lopez‐Munguia et al. 2011; Pavithra and Vadivukkarasi 2015; Senthil et al. 2015). After dose‐response relationships were confirmed at each drink, 100 μL of each of the test samples (pure water, AFS, AF, and B) was mixed with 1000 μL of 0.5 mmol/L DPPH solution (Tokyo Chemical Industry, Tokyo, Japan) and 900 μL of 1/15 mol/L phosphate buffer. The mixture was shaken vigorously and left to stand for 20 min, at which point the absorbance at 517 nm was measured against a reagent blank. The experiments were made in duplicate, and the mean value was recorded for analysis.

### Statistical analysis

Results are presented as means ± SEM. One‐way repeated‐measures ANOVA was used to compare parameters at each baseline. Changes in each parameter were analyzed by two‐way (trial × time) repeated‐measures ANOVA. In the case of a significant F value, the Bonferroni correction was used for post hoc multiple comparisons. Relationships were assessed using Pearson's correlation. One‐way ANOVA was also conducted to compare antioxidant capacity in each test sample. All data were statistically analyzed using SPSS 14.0J (IBM SPSS Japan, Tokyo, Japan) and Excel Statistics 2015 (Social Survey Research Information, Tokyo, Japan). Effect size (ES: using Cohen's *d*) and statistical power (1 − β) were calculated using G*Power 3. Differences were considered significant at *P* < 0.05.

## Results

**Figure 2:**
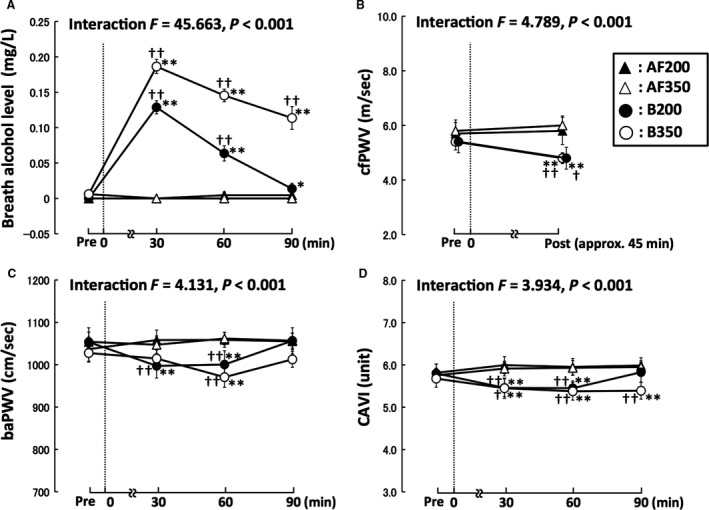
Effects of ingesting a small amount of beer on breath alcohol level (A), cfPWV (B), baPWV (C), and CAVI (D). Broken lines, time point of each beverage ingestion; Pre, baseline; cfPWV, carotid‐femoral pulse wave velocity, which is the gold‐standard indicator of central arterial stiffness; baPWV, brachial‐ankle pulse wave velocity, which reflects systemic arterial stiffness; CAVI, cardio‐ankle vascular index of arterial stiffness (adjusted for blood pressure); AF200 and AF350, alcohol‐free beer ingestion at 200 and 350 mL, respectively; B200 and B350, beer ingestion at 200 and 350 mL, respectively; *P < 0.05 versus each pre; **P < 0.01 versus each pre; †*P* < 0.05 versus each AF trial; ††*P* < 0.01 versus each AF trial. Data are expressed as mean ± SE.

Two‐way repeated‐measures ANOVA revealed significant interactions in BAC, cfPWV, baPWV, and CAVI (Fig. 2). No significant differences across baseline values of each trial were observed in all the parameters. BAC in AF200 and AF350 did not change throughout the experimental period, but BAC in B200 and B350 increased significantly at 30, 60, and 90 min postbeer ingestion (Fig. 2A). The results of estimated circulating alcohol levels also showed the same changes in BAC. Interestingly, cfPWV was significantly reduced following beer ingestion in the B200 (ES = 0.97, 1 − β = 0.95) and B350 (ES = 1.33, 1 − β = 0.96) trials, but not in the AF200 and AF350 trials (Fig. 2B). Mean baPWV and CAVI values were reduced only in the B200 (baPWV, 30 min ES = 1.16, 1 − β = 0.95; 60 min ES = 0.89, 1 − β = 0.96; CAVI, 30 min ES = 1.24, 1 − β = 0.96; 60 min ES = 1.03, 1 − β = 0.95) and B350 (baPWV, 30 min ES = 0.34, 1 − β = 0.95; 60 min ES = 0.74, 1 − β = 0.95; CAVI, 30 min ES = 0.72, 1 − β = 0.96; 60 min ES = 0.91, 1 − β = 0.95) trials during approximately 60 min postingestion (Fig. 2C,D). The reduced CAVI returned to the baseline levels at 90 min postingestion (a significant change) in the B200 trial (ES = 0.0), but not in the B350 trial (ES = 0.96, 1 − β = 0.96) (Fig. 2D). The estimated circulating alcohol levels were significantly and negatively correlated with the rate of changes in cfPWV (*r* = −0.54, *P* = 0.047, ES = 0.58, 1 − β = 0.78) and CAVI (*r* = −0.25, *P* = 0.047, ES = 0.25, 1 − β = 0.65) among 30, 60, and 90 min, but not baPWV (*r* = −0.083, *P* = 0.503). However, the change in arterial stiffness did not significantly correlate with the amount of beer consumed per body mass (cfPWV: *r* = 0.211, *P* = 0.468; baPWV 30 min: *r* = 0.318, *P* = 0.149; baPWV 60 min: *r* = −0.069, *P* = 0.761; CAVI 30 min: *r* = 0.119, *P* = 0.597; CAVI 60 min: *r* = 0.159, *P* = 0.481).

Table 1 shows the changes in hemodynamic parameters for each trial. No significant differences were observed across HR or BP at baseline. HR decreased slightly in both AF trials, but did not change significantly in either the B200 or B350 trials. Systolic BP, diastolic BP, and mean BP in the B200 and B350 were not significantly altered at 30 or 60 min postingestion. Diastolic BP in the B200 and B350 trials increased slightly after 90 min, but it did so in the AF200 and AF350 trials as well.

**Table 1:** Changes in hemodynamic parameters

Variables	Baseline	30 min	60 min	90 min	Main effect (trial)	Main effect (time)	Interaction
*Heart rate, beats/min*							
AF200	60 ± 3	51 ± 5^a^	55 ± 2^a^	51 ± 4^a^	*F* = 0.706	*F* = 3.977	*F* = 3.453
AF350	59 ± 3	55 ± 2^a^	55 ± 2^a^	54 ± 3^a^			
B200	57 ± 2	58 ± 2	56 ± 2	60 ± 2	*P* = 0.554	*P* = 0.014	*P* < 0.01
B350	59 ± 3	61 ± 3	60 ± 2	59 ± 2			
*Systolic BP, mmHg*							
AF200	120 ± 3	122 ± 2	122 ± 3	123 ± 2	*F* = 0.555	*F* = 0.228	*F* = 0.589
AF350	125 ± 2	125 ± 2	126 ± 3	125 ± 3			
B200	124 ± 3	122 ± 3	122 ± 3	122 ± 3	*P* = 0.648	*P* = 0.877	*P* = 0.804
B350	123 ± 2	125 ± 2	124 ± 3	123 ± 3			
*Diastolic BP, mmHg*							
AF200	69 ± 1	73 ± 1^a^	74 ± 1^a^	74 ± 1^a^	*F* = 0.343	*F* = 8.024	*F* = 1.814
AF350	72 ± 2	74 ± 2	73 ± 2	74 ± 2			
B200	71 ± 2	68 ± 1	72 ± 2	74 ± 2^a^	*P* = 0.794	*P* < 0.01	*P* = 0.105
B350	71 ± 2	71 ± 1	70 ± 1	76 ± 4^a^			
*Mean BP, mmHg*							
AF200	87 ± 2	91 ± 1^a^	91 ± 1^a^	93 ± 1^a^	*F* = 0.151	*F* = 5.864	*F* = 1.844
AF350	89 ± 2	91 ± 2	91 ± 2	92 ± 2			
B200	90 ± 2	88 ± 2	89 ± 2	92 ± 2	*P* = 0.928	*P* < 0.01	*P* = 0.067
B350	89 ± 2	91 ± 1	89 ± 2	90 ± 2			
*Pulse Pressure, mmHg*							
AF200	51 ± 3	48 ± 2	48 ± 2	48 ± 1	*F* = 1.817	*F* = 2.099	*F* = 0.911
AF350	53 ± 2	52 ± 2	53 ± 2	51 ± 2			
B200	53 ± 2	54 ± 2	50 ± 2	50 ± 2	*P* = 0.160	*P* = 0.120	*P* = 0.502
B350	53 ± 2	55 ± 2	54 ± 2	52 ± 1			

Data are expressed as mean ± SE.

BP, blood pressure; AF200 and AF350, alcohol‐free beer of 200 mL and 350 mL ingestion, respectively; B200 and B350, beer of 200 mL and 350 mL ingestion, respectively.

a *P* < 0.01 versus each baseline.

**Figure 3:**
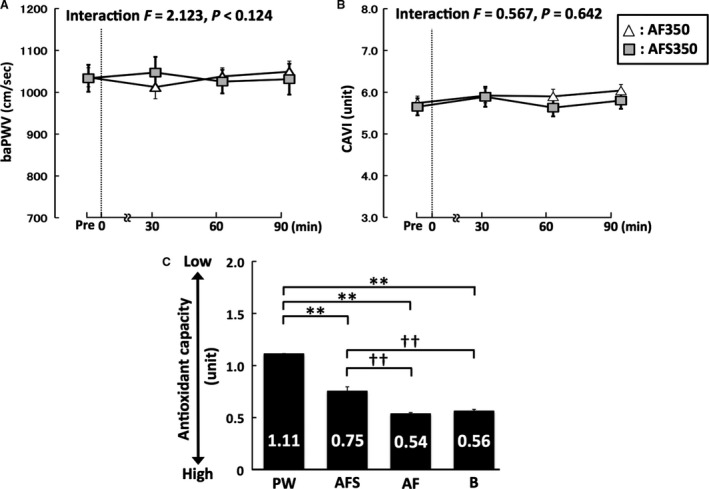
Supplementary experimental data for baPWV (A), CAVI (B), and antioxidant capacity (C). Broken lines, time points of each beverage ingestion; Pre, baseline; AF, alcohol‐free beer without sugar; AFS, alcohol‐free beer with sugar; PW, pure water; B, beer; baPWV, brachial‐ankle pulse wave velocity; CAVI, cardio‐ankle vascular index; **P < 0.01 versus PW; ††*P* < 0.01 versus AFS. Data are expressed as mean ± SE.

Figure 3 shows the results of supplementary experiments. In supplementary experiment 1, baPWV and CAVI did not change significantly in participants given alcohol‐free beer (in AF and AFS trials), regardless of whether it contained sugar (Fig. 3A,B). In supplementary experiment 2, antioxidant capacities were significantly higher in AF (ES = 7.3, 1 − β = 1.00) and B (ES = 6.1, 1 − β = 1.00) than in pure water or AFS. However, the antioxidant capacities did not significantly differ between AF and B (Fig. 3C).

## Discussion

The salient findings are that beer ingestion of 200 and 350 mL increases BAC and reduces cfPWV, baPWV, and CAVI. To the best of our knowledge, this is the first study to clarify the effects of ingesting only a small amount of beer on arterial stiffness.

In our experiments, because there was no food intake and only a small amount of fluid ingestion, PWVs and CAVI did not change significantly throughout the dealcoholized‐beer control trials, which is in contrast to a previous report (Karatzi et al. 2013). Nevertheless, cfPWV, baPWV, and CAVI were significantly reduced following ingestion in the B200 and B350 trials, and these reductions were sustained until at least 60 min postingestion. Interestingly, the changes in cfPWV were −0.6 ± 0.2 m/sec (−10.5 ± 4.0%) in B200 and −0.6 ± 0.2 m/sec (−10.1 ± 2.5%) in B350, and the reductions were almost the same between the present and previous studies despite the smaller volumes of beer ingested in this study (Karatzi et al. 2013). While cfPWV and baPWV are widely identified as the gold‐standard methods for assessment of arterial stiffness (Vlachopoulos et al. 2010, 2012), CAVI is also an index of arterial stiffness from the aorta to the ankle, after adjustment for BP, which is a major confounding factor (Shirai et al. 2011; Nishiwaki et al. 2014); a BP‐adjusted reduction in CAVI was also observed. In addition, no significant changes in BP and HR were observed 60 min after ingestion in either the B200 or the B350 trials, suggesting that these factors were not involved in the reductions of PWVs and CAVI, but rather, that these reductions truly reflect changes in arterial stiffness. Taken together, our findings indicate that ingestion of a small amount of beer induces acute reduction in arterial stiffness for approximately 60 min.

Beer contains alcohol, antioxidant substances, and sugar, and therefore, in this study, we investigated which of these components explain the acute reduction in arterial stiffness induced by beer ingestion. First, in Supplementary Experiment 1, we demonstrated that no significant changes in PWV and CAVI were observed in participants given alcohol‐free beer, regardless of whether it contained sugar. Thus, the effects of sugar in beer are considered to be negligible. Next, in the DPPH‐free radical scavenging activity analysis (Supplementary Experiment 2), antioxidant capacity did not differ between the alcohol‐containing beer and alcohol‐free control beer. Thus, antioxidant capacity seems unlikely to affect the reduction in arterial stiffness. Finally, ingestion of even a small amount of beer, such as 200 or 350 mL, increased BAC, and the reduction in arterial stiffness was found only when a 0.05 mg/L increase in BAC was observed. Moreover, the circulating alcohol levels were significantly and negatively correlated with the changes in cfPWV or CAVI as indexes of arterial stiffness. Therefore, our data suggest that the alcohol in beer is the main contributor to the reduction in arterial stiffness associated with beer consumption.

How the beer or alcohol reduces arterial stiffness remains unknown. However, the changes in arterial stiffness are generally thought to results from structural changes (i.e., elastin and collagen content), functional changes (i.e., sympathetic nervous activity, vasoactive substances), or a combination of both (Tanaka et al. 2000; Tanaka and Safar 2005). Because structural changes in the elastin‐collagen composition of the arterial wall are believed to occur over weeks or years (Tanaka et al. 2000; Tanaka and Safar 2005), an acute change in arterial stiffness following alcohol ingestion is probably mediated by an alteration of functional changes (Tanaka et al. 2000; Sugawara et al. 2004; Tanaka and Safar 2005). In the present study, BP and HR did not decrease significantly and/or increased slightly after acute beer ingestion. However, a previous study has indicated that acute ingestion of alcohol reduces BP variability, which is index of sympathetic control of vasomotor tone (Buckman et al. 2015). Thus, one possible mechanism underlying acute effects of beer or alcohol ingestion on arterial function may be related to the change in sympathetic control of vasomotor tone. On the other hand, studies in vitro have demonstrated that a low concentration of alcohol can promote nitric oxide (NO) release from endothelium through upregulation of NO synthase, while high concentrations or chronic consumption of alcohol could impair endothelial function by decreasing NO bioavailability (Toda and Ayajiki 2010; Zhou et al. 2016). NO, as an important endogenous vasodilator, plays a key role in regulating blood pressure and protecting against pathological vascular damage (Toda and Ayajiki 2010). Therefore, it is also possible that a small amount of beer or alcohol could promote an increase in NO, thereby reducing arterial stiffness. Relevant to this, beer ingestion was recently shown to increase vascular endothelium function (Tousoulis et al. 2008; Karatzi et al. 2013).

Is regular ingestion of a small amount beer or other alcohol beverages associated with a lower risk of cardiovascular and all‐cause mortalities? Epidemiological studies have demonstrated that J‐shaped relationships are observed for cardiovascular disease and/or all‐cause mortalities (Arranz et al. 2012; de Gaetano et al. 2016; Zhou et al. 2016). However, longitudinal studies indicate no significant relationship between alcohol consumption and diseases, and relative risks for mild drinkers were approximately the same as for nondrinkers (light drinkers 1.08 vs. nondrinkers 1.00) (Nakanishi et al. 1998, 2001). In addition, the ingestion of alcoholic beverages in excess of the mild‐to‐moderate level is known to elicit a reduction in arterial compliance, which means an increase in arterial stiffness (Fantin et al. 2016). Thus, vascular responses can differ acutely according to the amount of alcohol consumed. Also, although the previous studies have already reported acute reduction in arterial stiffness after beer ingestion (Krnic et al. 2011; Karatzi et al. 2013), the current study has several strengths and new findings. That is, data from the present study provide support for the positive effects on a reduction in arterial stiffness of ingesting a small amount of beer, corresponding to the daily intake of mild drinkers, and suggest that the alcohol in beer is the main contributor to the acute reduction in arterial stiffness. Therefore, to elucidate whether regular beer or alcohol consumption reduces arterial stiffness, further interventional and longitudinal studies are required in terms of quantity and duration of ingestion.

There are a few potential limitations to this study. First, previous experimental studies show two types of methods for alcohol intake: absolute amounts or body mass‐adjusted relative amounts (Krnic et al. 2011; Karatzi et al. 2013; Fantin et al. 2016). Because recommended alcohol intake is defined by absolute amounts and we intended for the results of our study to be easily generalizable to clinical settings, participants were given two different relatively small absolute amounts of beer (200 and 350 mL); however, changes in arterial stiffness did not significantly correlate with relative amounts per body mass. Accordingly, it seems unlikely that the reduction in arterial stiffness were profoundly affected by the minor differences in mass‐based relative ingesting amounts of each participant. Second, the results of DPPH indicate antioxidant capacity in vitro, and antioxidant capacity in vivo should be assessed by physiological parameters after the ingestion. In this study, such parameters did not measure, but it is the most important that antioxidant capacity did not differ substantially between beer and alcohol‐free beer, which were utilized in this study. Accordingly, although further studies are needed to clear the points of physiological parameters, our data strongly suggest that the reduction in arterial stiffness is mainly attributable to the effects of the alcohol in beer. Also, other factors not addressed in this study, such as consumption of other types of alcoholic beverages (e.g., wine or spirits), sex, heavy drinking, older age, or preexisting arterial stiffness would likely affect the acute changes in arterial stiffness induced by ingestion of small amounts of beer. Thus, our results showing that a small amount of beer ingestion reduces arterial stiffness are specific to young, healthy, male, mild‐to‐moderate drinkers. Therefore, additional investigations using different protocols and study populations may reveal important new insights into the relationship between arterial stiffness and alcohol ingestion.

In conclusion, our results indicate that ingesting a small amount of beer induces an acute reduction in arterial stiffness for approximately 60 min. Our data also suggest that this reduction in arterial stiffness is mainly attributable to the effects of the alcohol in beer. These findings could therefore offer new insights into vascular biology, as well as important implications for development of a new effective method for preventing arterial stiffening.

## Conflict of Interest

The authors declare that there is no conflict of interests regarding the publication of this article.
